# Between-Session Reliability of Athletic Performance and Injury Mitigation Measures in Female Adolescent Athletes in the United States

**DOI:** 10.3390/life14070892

**Published:** 2024-07-18

**Authors:** Emily Franklin, Georgina K. Stebbings, Christopher I. Morse, Adam Runacres, Thomas Dos’Santos

**Affiliations:** Department of Sport and Exercise Sciences, Manchester Institute of Sport, Manchester Metropolitan University, Manchester M1 7EL, UK; emily.franklin@stu.mmu.ac.uk (E.F.); g.stebbings@mmu.ac.uk (G.K.S.); c.morse@mmu.ac.uk (C.I.M.); a.runacres@mmu.ac.uk (A.R.)

**Keywords:** repeatability, youth, girl, strength, power, speed, post-PHV

## Abstract

Adolescence is a fundamental period for female athletes to develop athletic performance, mitigate injury risk, and gain collegiate sport scholarships, but there is also a high incidence of sport-related injuries. Physical profiling and athlete screening can support the individualisation of training programmes; however, there is a lack of data pertaining to the reliability of athletic performance and injury surrogate measures in adolescent female athletes. The aim of this study was to quantify the between-session reliability of an athletic performance and injury mitigation testing battery in female adolescent athletes. A total of 31 post-peak height velocity (PHV) (3.00 ± 0.82 years) female athletes (age: 16.20 ± 1.20 years; standing height: 166.00 ± 6.00 cm; mass: 65.5 ± 10.70 kg) from various sports (track and field = 1; lacrosse = 2; basketball = 2; soccer = 3; softball = 11; volleyball = 12) completed two sessions of a multicomponent testing battery 48 h to 1 week apart including the assessment of 33 measures addressing lower-limb isometric strength, eccentric strength, reactive strength, linear sprint and change of direction speed, and lower limb control. Of the 33 measures, between sessions, 29 had a high to nearly perfect intraclass correlation coefficient (ICC) (0.508–0.979), and 26 measures were not statistically significantly different between sessions (*p* ≤ 0.05). All measures demonstrated low to acceptable coefficient variation (CV%) (0.61–14.70%). The testing battery used can be utilised for recruitment and longitudinal monitoring within sports organisations for female adolescent athletes.

## 1. Introduction

Following the passing of Title IX in the United States (US) which promoted gender equality in sports [1], in addition to the Amateur Sports Act of 1978 which prohibited discrimination in amateur level sports [2], female sports participation has increased from 1 in every 27 girls in 1972 to 1 in every 3 girls in 1998 within the US [3]. Concerningly, female athletes have been observed to experience a greater number of injuries compared to their male counterparts [1]. For example, female athletes were 2–4 times more likely to experience an injury compared to males [4], with the injuries predominately occurring in the knee [1]. The age at which injury occurrence peaked for female athletes is reportedly 16 years old [5]. In the US, 16 years is the age at which collegiate sports coaches are permitted to contact and scout potential candidates for their university [6]. It could be implied that with recruitment for collegiate athletic opportunities and scholarships potentially starting in the first or second year of high school (15–16 years old), female athletes wanting to secure a scholarship or be recruited to play a sport will aim to achieve the necessary collegiate level for key athletic performance indicators in readiness for the start of the formal recruiting process. Actions for achieving such goals include increases in training and academic workloads [7]. 

The process to assess key athletic performance indicators, and potential talent identification, is completed via fitness testing or physical profiling [8]. This process is adopted by practitioners to assess the physical capabilities of athletes such as speed, strength, and power through a testing battery [9]. Indicators of performance can either be assessed qualitatively via a grading scale or more accurately, reliably, and objectively through quantitative measures using sport technology such as force platforms, strain gauges, timing cells, and cameras [10]. The information collected from fitness testing can be used to create athlete benchmarks and normative data, but more importantly, it can provide insight into areas of strength and improvement to aid in the individualised development of the athlete in their respective sport [9]. In addition to fitness testing and physical profiling, another method of athlete assessment is to screen for injury risk surrogates (i.e., metrics which contribute to high mechanically induced tissue damage or are associated with potential injury risk) [11]. Some potential injury risk surrogates include knee valgus (i.e., ligament dominance) when landing which can increase knee mechanical loading [12] and subsequent anterior cruciate ligament (ACL) injury risk and patella femoral pain (PFP). Due to the injury risk being greater in female athletes, screening for surrogate measures and implementing injury prevention programmes is essential to prolonging their athletic career and maximising their athletic potential [13]. 

Injury screening methods such as the landing error scoring system (LESS) and 2D motion analysis to attain the frontal plane projection angles (FPPAs) are used in athlete testing [14,15]. The LESS protocol qualitatively grades an individual’s landing mechanics [14], with higher scores representative of greater knee injury risk, while frontal plane projection angles measured using 2D motion analysis indicate the degree to which the knee medially moves towards a valgus position [15] and can be measured during unilateral (i.e., single leg squat) [16] and bilateral tasks (i.e., rebound jump). Furthermore, the tuck jump assessment has been used to assess movement quality linked to potential ACL injury via a grading scale that assesses landing mechanics such as knee valgus [17]. Knee valgus increases the moment arm in the frontal plane and thus the knee abduction moment and subsequent knee mechanical loading. Read et al. [12] composed a grading system to aid practitioners and coaches when classifying an athlete’s frontal plane projection angle into a minor (≤10°), moderate (10–20°), or severe (≥20°) classification. It should be noted that Read et al. [12] created this classification system in elite youth male participants which may not be fairly translated to female participants. According to Emmonds et al. [18], research regarding sporting practices such as injury prevention protocols can be considered limited in terms of female representation, and sub-optimal in methodological and research quality, and thus a recommended area for future research.

In an athlete’s sporting career, their performance and risk of injury can be assessed multiple times in a calendar year at various stages of the season. The purpose for multiple testing periods throughout the year is to monitor the acute and chronic effects of training interventions [9], or to establish the presence of potential fatigue. However, in order to establish whether “real” and “meaningful” changes in performance and injury risk surrogates have occurred, the data must be reliable. Between-session reliability (i.e., between day test–retest/inter-session) has been deemed essential to allow coaches and practitioners to monitor potential changes within the athlete [19]. Thomas et al. [9] defined reliability as the consistency of the testing measure, which is unique to the testing procedures adopted and is population-specific. The between-day reliability of a test refers to its ability to produce consistent results from day-to-day [19]. This method of assessing athletes allows coaches to be confident that the changes in performance from a specific test are “real” and not due to daily variations in the test; thus, it is important that the test has good between-day reliability.

During adolescence, which is approximately 11–19 years old for girls [20], girls experience accelerated skeletal growth and body composition changes such as fat redistribution [21]. Such changes can alter the whole body and segmental centre of mass and bodily mass, affecting limb lengths and potential lever arms (i.e., greater torques), thus affecting motor control and potentially increasing the mechanical load for an adolescent female [22]. The aforementioned physiological and biomechanical changes are paired with the disproportionate change in body composition and strength, which are potential factors that may increase injury risk [23]. Hence why practitioners should monitor development and assess reliability of key fitness and screening measures in girls during adolescence, particularly post-peak height velocity (PHV). Currently, there is a limited number of studies that have examined the between-session reliability of neuromuscular performance and injury surrogate measures in adolescent athletes, particularly in the US. Of the few studies that have examined between-session reliability in adolescent females, high reliability has been observed such as Moeskops et al. [24] who established an almost perfect reliability (CV%: 5.8–6.7-; ICC: 0.92–0.95) for the isometric mid-thigh pull for post-PHV in female athletes. McCubbine and Turner [25] established sufficient reliability for the single leg triple hop distance in elite youth (10–11 years old) female footballers (CV%: 3.3 and 3.6; ICC: 0.85 and 0.87) for the left and right leg. It should be noted that the definition of elite by McCubbine and Turner [25] was athletes from a professional soccer club. Sawczuk and Jones [8] established high reliability in the countermovement jump, jump heights (CV%: 2.8), 505 completion times (CV%: 4.1; 5.4), and 40 m sprint completion time (CV%: 1.8) in a mixed youth sample. Moreover, Cuthbert et al. [26] established an almost perfect reliability in Nordic eccentric hamstring strength via a Nordbord within senior professional female soccer players (17–25 years old) (ICC: 0.901), but to the authors’ best knowledge, there is no research regarding reliability for assessing hamstring strength via the Nordic hamstring exercise within female adolescent athletes. The methodological differences within these studies with regard to sex and age does not fully represent the growth and maturation of adolescence, thus raising concerns for application to late adolescent athletes.

The lack of female representation in research is perhaps exacerbated by a tendency to apply existing research in male adolescent athletes rather than female adolescent athletes without consideration of the well-documented divergence in physical characteristics and athletic abilities according to sex during adolescence. Therefore, the aim of this study was to develop and quantify the between-session reliability of a physical profiling testing battery in American female adolescent athletes. It was hypothesised that the vertical countermovement jump, isometric mid-thigh pull, linear sprinting, 505 change of direction speed, and Nordbord measures would produce high and acceptable between-session reliability. Moreover, it was further hypothesised that high and acceptable between-session reliability would be observed for injury surrogate measures during the single leg squat and tuck jump assessments.

## 2. Materials and Methods

### 2.1. Experimental Design

This study assessed the between-session reliability of a physical performance and injury risk profiling testing battery using a within-subject repeated measures design. The participants performed two testing sessions 48 h to 1 week apart (to allow for adequate recovery time), with both sessions occurring at the same time of day for each participant (either morning, mid-afternoon, or evening) to minimise the impact of circadian rhythm [27]. Hereafter, the first testing session will be termed “session 1” with the second day of testing termed “session 2”. The participants were instructed to maintain similar diet, sleep, and daily activities between sessions while refraining from physical activity for 48 h prior to the testing sessions. All testing took place at the Pro Motion Performance facility (Minooka, IL, USA) and all tests took place on a rubber surface. Athletes were advised to wear shorts or leggings, tops they were able to tuck in, and the same shoes to both sessions for consistency and screening purposes. The ethics committee of Manchester Metropolitan University approved the research (Ethos ID: 54450; project ID:850160; and date of approval: 17 May 2023).

### 2.2. Participants

Participants were recruited from any sport, organisation, and all competition levels above recreational, here defined as an athlete who has participated in competitive sport. Recruitment was undertaken at local sports clubs, traveling amateur-level teams, and high schools in the area. An a priori power was calculated (80% power, expected ICC = 0.90, *p* ≤ 0.05) [28] with a 10% drop out which deemed the minimum sample size as 23 participants (26 with 10% drop rate). In total, 31 adolescent, cisgender, female athletes (age: 16.20 ± 1.20 years; standing height: 166.00 ± 6.00 cm; sitting height: 84.60 ± 3.40 cm; mass: 65.5 ± 10.7 kg) from various sports (track and field = 1; lacrosse = 2; basketball = 2; soccer = 3; softball = 11; volleyball = 12) were recruited for this study. According to McKay et al.’s [29] athlete tier system, 30 of the athletes were classified as Tier 2 and one was classified as Tier 1. Tier 1 is defined as individually participating in sport one to two times per a week and Tier 2 is defined as individuals participating in a sport organisation at the local level, attending organised training sessions three times per week minimum [29]. The maturation status was calculated via Mirwald et al.’s [30] maturation offset equation and all participants were classed as post-PHV (3.00 ± 0.82 years). The inclusion criteria were as follows:

(a) Have 1–6 years of experience playing their sport competitively;
(b) Injury free for the 6 months prior to the start date of the study;
(c) Are 14–18 years of age;
(d) Cisgender individuals born biologically female;
(e) Understand written and spoken English.

Individuals who fell outside of the permitted age range, were male or identified as a transgender man, played sports at a recreational level, and/or had the inability to understand written and/or spoken English were not included in the study. At the time of testing, the participants were either in-season or within their preseason phase in their respective sports (in season = 18; preseason = 13). The participants were asked their limb preference for kicking (right *n* = 23; left *n* = 8), throwing (right *n* = 29; left *n* = 2), and turning to change direction (right *n*= 23; left *n* = 8). All participants provided informed consent or parental assent (if under 18 years old) prior to participating in the testing.

### 2.3. Protocol and Procedures

#### 2.3.1. Warm Up

Before completing the testing battery, the participants completed a standardised 10 min dynamic warm-up following the RAMP protocol (raise, activate, mobilise, potentiate) (Table 1) that complied with NSCA protocols [31].

##### Testing Procedure

Upon the athlete’s arrival to the facility, anthropometrics were collected to estimate maturation status. Following the warm-up, 2D analysis injury screening was completed first before randomly assigning the athletes to different testing stations and completing the force platforms, hamstring/knee flexor strength, and triple hops for distance in a cyclical manner before finishing with linear sprints and change of direction agility (standardised between-sessions). This order was to promote testing efficiency to accommodate the moderate to large groups (6–12 athletes per session) coming to the facility. Rest periods (3–5 min) were provided between tests for recovery and to provide instruction for the following test, and the overall testing per a participant lasted approx. 1–1.5 h.

#### 2.3.2. Anthropometrics (Estimating Biological Age)

To assess the maturity of the participants, the study utilised peak height velocity (PHV) assessments and maturity off-set. The assessment involved measuring standing and sitting height via a stadiometer (Charder Medical, HM200P, Taichung, Taiwan), body mass via a digital scale (Etekcity, Anaheim, CA, USA), and date of birth. This was performed by instructing the participant to take off their shoes and stand with their back against the stadiometer to collect their standing height then to L-sit in the base of the stadiometer and to sit as straight as possible to obtain the sitting height measurement before the athletes stood on the digital scale to obtain their mass [32]. Maturity status was evaluated via the thresholds pre-PHV (offset ≤−1 years), circa-PHV (offset between −1 and +1 year), and post-PHV (offset ≥+1 years) to provide a calculated difference in the estimated skeletal age relative to chronological age [32,33]. Maturity offset was derived using Microsoft Excel spreadsheet developed by Towlson and Salter [34].

#### 2.3.3. Neuromuscular Performance

##### Force Platform Testing Preface

The bilateral isometric mid-thigh pull (IMTP) and bilateral vertical countermovement jump (CMJ) were each performed on two sets of portable dual force platforms (Hawkin Dynamics, 5th generation, controller version 4.3.3, Westbrook, ME, USA), sampling at a frequency of 1000 Hz. Prior to each of the three trials, the force platforms were zeroed to reduce signal noise. Vertical ground reaction forces (GRFs) during the IMTP and CMJ were recorded and all data regarding performance and injury metrics stated below were obtained and calculated via Hawkin Dynamics proprietary software on the issued Lenovo tablet (Lenovo P11 Pro, 2nd generation) and stored in the Hawkin Dynamics cloud database. Key outcome measures were then exported into a Microsoft Excel for subsequent statistical analysis. Raw vertical GRFs were filtered through a low pass 50 Hz cut-off frequency via Hawkin Dynamics proprietary software. 

##### Bilateral Isometric Mid-Thigh Pull (IMTP)

The IMTP is a method of assessing neuromuscular strength (i.e., rapid and maximal force production), and has been deemed to be more time efficient, safer, and less fatiguing than 1 repetition max testing, especially in large group settings [35,36] and has been implemented safely in female adolescent athletes [37]. Using the abovementioned portable force platforms and a portable isometric rig (Hawkin Dynamics, ME, USA), whereby the bar height could be adjusted at 3 cm increments, the participants performed a bilateral IMTP in line with standardised and recommended guidelines [38]. The participants were asked to adopt their preferred power position that reflects the start of the 2nd pull of a clean [38]. Due to the inter-individual variation in anthropometrics and movement competency levels, the study utilised joint angle configurations deemed acceptable at the knee and hip joint (120–135°/140–150°), within their preferred power position for the starting position, as used in a previous study [35], and these were standardised for longitudinal comparisons. 

Prior to the pull, the participants were instructed to take off their shoes, place their feet approximately hip width apart, having their mid-foot cover the logo that is located centrally on the force platforms (directly underneath the bar), hands slightly wider than shoulder width with an over-hand grip, shoulder blades retracted, and shoulder joints over the bar. The bar was adjusted to align with the upper thigh before the participants were strapped onto the bar using weightlifting straps (Harbinger Fitness, Duragrip, Carlsbad, CA, USA). Once the participant was in position, they were instructed to stand as still as possible to collect a one-second silent weighing period, and on a “GO” command, to then pull the bar and push their feet directly downwards into the ground as fast and as hard as possible for 3–5 s, with the cue “push, push, push” during their attempt [38]. If the participant performed a dynamic start (i.e., countermovement) at the beginning of their attempt or released the bar during the pull, this trial was not counted and another trial was performed following a 2 min rest period. For this assessment, peak force, initiation threshold (calculated as 5SD of the 1 s weighing period) [38], and gross force at time intervals of 50, 100, 150, 200, and 250 ms were collected. The participants were given 2 warm up trials (50% and 75% of maximum perceived effort) before they performed 3 maximal effort attempts with 2 min of rest between each attempt [24]. 

##### Vertical Countermovement Jump

A bilateral vertical countermovement jump (CMJ) was used to assess the reactive strength and slow stretch shortening cycle function of the participants [39]. This was performed on the force platform mentioned previously, with the participants instructed to step onto the force platform before standing as still as possible to collect a silent weighing period and then instructed to “jump vertically as fast and as high as possible” when a “GO” command was given. The participants performed the jump with arms akimbo and were instructed to maintain extended lower limbs during the flight phase before cushioning the landing upon contact with force platforms. Badby et al. [40] determined that the Hawkin Dynamics force platform is a valid method of obtaining CMJ metrics. The participants performed two warm up trials before performing 3 maximal effort trials with 2 min of rest between trials.

The following metrics were examined and calculated using Hawkin Dynamics proprietary software: jump height, time to take off, jump momentum, reactive strength index modified, displacement, landing forces (peak and average), time to stabilise, and landing stiffness. The metrics and definitions provided by Hawkin Dynamics for the aforementioned tests are provided in Table 2.

#### 2.3.4. Triple Hops for Distance

For insights into horizontal impulsive capabilities, the final jump assessment was triple, single leg hops for distance on a rubber, indoor track. The protocol followed previous studies by Trigsted and Post [41] and McCubbine and Turner [25] and horizontal distance was measured via a laser measure (Dtape, DT50, Shenzhen, China) perpendicular to the start line to the back of the designated heel. The participants were instructed to perform an initial countermovement and hop three times consecutively on each leg (hands akimbo to isolate the lower-limb contribution) before sticking the landing by standing as still as possible during measuring until the participants were instructed to move. If the participants landed with both feet within the three jumps, could not stabilise the landing for more than two seconds, or released their hands from their hips during the jumps, it resulted in a restart of the trial [42]. After two familiarisation trials, the participants performed three trials on each leg in a counterbalanced order. 

#### 2.3.5. Hamstring/Knee Flexor Strength

A Vald Nordbord (Brisbane, QLD, Australia), sampling at 50 Hz, was used to assess eccentric hamstring strength. Participants were instructed to perform a Nordic curl (an eccentric hamstring muscle action, from a kneeling position, with the ankles secured in place) while maintaining a neutral back and descending to the floor as slowly as possible while maintaining hip extension. Athletes were instructed to “lock” their ankles into the individual hooks of the device, maintain a 90° angle of the ankle and knee in relation to the equipment, knees hip-width apart, and have hands slightly forward to catch themselves. Padding was placed in front of the equipment for additional safety measures. If the athlete hinged at the hip, came out of the hooks, or caught themselves too early, a restart was required after the specified rest period below. After two familiarisation trials to establish technique and procedure standards, participants performed three trials with two minutes rest between trials where the peak force of each leg were quantified [43]. Forces for each leg was assessed by the Vald proprietary software and exported into an excel spreadsheet. Total force (i.e., the sum of the peak force on each leg) was calculated during each trial. 

#### 2.3.6. 2D Analysis

The purpose of this analysis was to assess the landing mechanics, neuromuscular capacity, and movement quality of the participants [44]. One Teledyne FLIR camera (1.6 MP B&W Blackfly S USB 3.0, Wilsonville, OR, USA) was placed in the frontal plane on a tripod (0.75 m high) and three metres away from the marked area to video record the movements mentioned below. Spinnaker software (Teledyne FLIR, Wilsonville, OR, USA) was used to obtain recorded videos of the movements mentioned below. Additionally, reflective tape was placed by the principal investigator on the anterior superior iliac spine mid-thigh, centre of the patella, and centre of the ankle (frontal plane) on the participant for each lower limb [44]. The frontal plane projection angle (FPPA), a proxy for knee valgus and subsequently knee mechanical loading associated with ACL injuries and patellofemoral pain [15,44], was measured during tasks using Quintic software (Version v33, Birmingham, England). For consistency purposes, the principal investigator determined the intra-rater reliability for each test to ensure consistent angle measurements. Intra-rater reliability assessment was conducted with 12 weeks between measures. The ICCs were observed to be nearly perfect (0.927–0.999) with trivial effect sizes for FPPA (*d* = −0.04–0.13), along with statistically not significant differences between the first and second sessions of the tuck jumps and single leg squats (*p* > 0.05).

##### Single Leg Squats (SLSs) and Tuck Jumps

The participants performed 5 single leg squats on each side with arms akimbo and the opposite leg at 90° knee flexion with the hip neutral and instructed to maintain their balance while avoiding placing their contralateral limb on the ground. The participants were further instructed to squat down to a depth where they were able to maintain stability eccentrically and concentrically while keeping markers in visible of the camera. Movement quality for performing a single leg squat was assessed prior to testing for all athletes. The degree of risk regarding ACL and other knee-related injuries via the FPPA were calculated using the software mentioned above [14,45]. The participants also performed a 10 s repeated tuck jump test to identify any additional injury surrogates such as quadriceps, trunk dominance, leg dominance, or knee ligament dominance [46]. The participants were instructed to keep arms akimbo, and in a rebounding motion, to jump bringing their knees as high as possible. If the participant let go of their hips, fell, or jumped out of the camera frame, a retrial was performed after two-minute rest period. The last three tuck jumps and single leg squats from each attempt were measured for FPPA using the Quintic software. A two-minute rest between each attempt was adopted following NSCA rest period recommendations for plyometric training [31]. 

#### 2.3.7. Multidirectional Speed Profiling

##### Linear Speed

The participants completed three trials of a 30 m linear sprint from a two-point staggered stance 0.3 m behind the starting point. Five sets of photocell timing gates (Witty, Bolzano, Italy) were placed along a rubber track (2.5 m width) set at 0 m, 10 m, 20 m, and 30 m at approximate hip height on a rubber, indoor track. A similar protocol has been used in a previous study [47] with adolescent female handball players. The timing gates were used to obtain split times at each distance marker at 10 m, 20 m, and 30 m. The participants completed two warm up trials (at 50 and 75% of perceived maximal effort) before completing the three maximal effort trials with three minutes of rest between attempts. 

#### 2.3.8. Change of Direction Speed

The participants performed three trials of a 505 test for both left and right directions. Two sets of cones were placed at the start and at 15 m with two sets of timing gates placed at the 0 and 10 m marks at approximately hip height on a rubber, indoor track. This test has been deemed a reliable test in adolescent to adult female athletes (ICC:0.968) [48]. The participants were instructed to sprint through the gates to the farthest cones where they touched the line with either the left or right foot (depending on trial), turned 180° off the designated leg then sprinted back 5 m through the 10 m timing gates again to finish [49]. If an athlete turned on the wrong foot, turned or stopped on the wrong line, and/or missed the timing gate, a retrial was required. The participants were given two minutes of rest based on previous literature [50].

## 3. Statistical Analysis

Testing session measures for dependent variables are presented as mean ± standard deviation (SD). All statistical analyses were performed in SPSS v 29 (SPSS Inc., Chicago, IL, USA) and Microsoft Excel (version 2016, Microsoft Corp., Redmond, WA, USA). Reliability measures including coefficient of variation (CV%) and standard error of measurement (SEM) were used to examine absolute reliability. However, as suggested by Bailey [51] for metrics which contain both negative and positive values (such as FPPA), only the SEM was calculated. Additionally, the smallest detectable difference (SDD) of the mean of sessions 1 and 2, and effect size (ES) were all calculated via Microsoft Excel. The calculations for reliability measures were as follows:

coefficient of variation (CV% = SD/mean × 100)(1)

(2) $$\mathrm{SEM}=(\mathrm{SD}\;(\mathrm{pooled})\;\times \;\sqrt{1-ICC})$$

(3) smallest detectible difference=1.96 × (√2) × SEM

Cohen’s *d* effect size = session 1 mean − session 2 mean/SD(pooled) (4)

Additionally, reliability variables, including intraclass correlation coefficient (ICC) (two-way mixed effects, average measures, absolute agreement) to examine relative reliability, and *p*-values were calculated via SPSS software and normality was assessed via the Shapiro–Wilk statistic in SPSS. SDD was used to determine real and meaningful changes between the sessions [52]. CV% and SEM were used to determine the variability between the two sessions. Paired sample *t*-tests were used to examine bias and compare dependent variables between testing sessions. For non-parametric data, a Wilcoxon sign ranked test was performed. ICCs were interpreted as follows: ≥0.9 almost perfect; 0.7–0.9 very high; 0.5–0.7 high; 0.3–0.5 moderate; 0–0.3 low [53]. Effect sizes (*d*) were classified as ≥4.0 extremely large; 2.0–4.0 very large; 1.2–2.0 large; 0.6–1.2 moderate; 0.2–0.6 small; ≤0.2 trivial [53]. Statistical significance was classified as a *p*-value ≤ 0.05 [24] and CV% was classified as ≤5% excellent; 5–10% good; 10–15% acceptable; ≥15% unacceptable [54].

## 4. Results

### 4.1. Between-Session Reliability

Descriptive statistics and reliability measures containing ICC, CV%, SEM, SDD, *p* values, and ES are presented for all assessments in Table 3, Table 4, Table 5 and Table 6. The Shapiro–Wilk normality test demonstrated that all IMTP measures excluding initiation threshold were normally distributed (*p* = 0.069–0.960). For CMJ measures, the Shapiro–Wilk test signified that the session 1 jump height and time to take off, session 2 jump momentum, propulsive impulse, landing stiffness, time to stabilise, average landing force, and peak landing force were not normally distributed (*p* < 0.05); all other measures were distributed normally (*p* = 0.054–0.828). Between-session IMTP ICCs were high to almost perfect (0.546–0.909), with acceptable to good CV% values observed (7.40–14.70%) (Table 3). There were no significant differences, with trivial effect sizes between sessions for initiation threshold and force at 50 ms; however, statistically significant increases in testing session 2 for forces at 150, 200, and 250 ms (Table 3) were observed. Between-session CMJ ICCs were deemed moderate for time to stabilise (0.476), and very high to almost perfect for all other CMJ measures (0.730–0.979) (Table 3). The CV% values were unacceptable for landing stiffness and time to stabilise (22.65 and 21.67%), acceptable for countermovement displacement and peak landing force (13.46 and 10.39%), and good to excellent for all other CMJ measures (CV < 10%; Table 3). There was no significant difference with trivial effect sizes observed between sessions for all CMJ metrics (Table 3) excluding countermovement displacement, time to take off, time to stabilise, and peak landing force, which demonstrated non-significant small differences between trials (ES: 0.21–0.32). Peak landing force was the only CMJ metric that reported a statistically significant difference between sessions (Table 3).

The eccentric hamstring strength and single leg hop data were normally distributed (all *p* > 0.05; Shapiro–Wilk: 0.081–0.362). All measures for both assessments exhibited almost perfect between-session ICC values (0.913–0.954) excluding left force for hamstring strength which demonstrated a very high ICC (0.881). The CV% values were classified as good for eccentric hamstring strength (6.30–8.28%) and excellent for the single leg triple hop (4.08 and 4.61%; Table 4). There was no significant difference between sessions for eccentric hamstring strength and single leg triple hop metrics, with trivial effect sizes (Table 4).

The session 1 and 2 sprint data were normally distributed (*p* = 0.128–0.369); however, the session 2 data were not normally distributed (*p* < 0.05) for the 20 m and 30 m sprint times. The between-session sprint ICC values were very high to almost perfect (0.809–0.926) with excellent CV% values for the 10, 20, and 30 m times (2.07–2.55%) (Table 5). There was no significant difference between sessions for the 10, 20, and 30 m sprint times with trivial effect sizes (Table 5).

Measures of the left 505 change of direction in session 1 exhibited a normal distribution; the 505 time for both sessions also exhibited a normal distribution with the only exception of the non-10 m approach in session 2. Very high to almost perfect ICC values and excellent CV% values were observed for all measures (ICC = 0.852–0.946; CV% = 1.80–2.37). There was no inter-session difference in the 10 m approach for either direction (Table 5). The right foot 505 measures did not exhibit a normal distribution, except for the session 1 505 time, and has high to very high ICC values (0.782–0.911). The CV% values for all right foot measures were excellent (2.23–2.96%). No statistically significant differences between sessions were observed for COD speed measures except for left foot 505 time (*p* < 0.05), with trivial to small effect sizes for all COD metrics (Table 5). 

Tuck jump and right single leg squat FFPA measures were normally distributed (*p* = 0.082–0.0878) except for left single leg squat FPPA (both sessions) and the between-session tuck jump assessment FPPA. The ICC values were high to very high (0.822–0.874), whereas the single leg squat FPPA ICC values were moderate to high (0.465–0.508) (Table 6). No statistically significant differences were observed between sessions for FPPA, with trivial to small effect sizes (Table 6). The intra-rater reliability results can be found in the Supplementary Materials.

The ICC and CV% 95% confidence intervals for all tests can be found in the Supplementary Materials.

### 4.2. Within-Session Reliability

Descriptive statistics of the within-session reliability measures containing mean, SD, ICC, CV%, and SEM are presented in the Supplementary Materials. Of the measures in session 1, 21 had an almost perfect ICC (0.901–1.001), 11 had a very high ICC (0.711—0.899), one had a high ICC (0.667) [time to stabilise]. A high absolute reliability regarding CV% was exhibited in session 1, where 25 measures ranged from excellent to good (0.05–9.04%), with 1 acceptable (10.01%) and 4 were unacceptable (15.17–28.53%). For session 2, the reliability results were slightly higher, with 20 measures exhibiting an almost perfect ICC (0.901–1.000), 10 had a very high ICC (0.711–0.899), and two had a high ICC (0.6667 and 0.698) [time to stabilise]. Similar reliability was observed for session 2 with 23 measures with a good to excellent CV% (0.09–9.02%), 4 with an acceptable CV% (10.02–12.83%), and 3 with an unacceptable CV% (20.56–24.20%). The results tables for within-session reliability can be found in the Supplementary Materials. 

## 5. Discussion

The primary aim of this study was to establish and quantify the between-session reliability of a physical profiling testing battery in American female adolescent athletes. The primary findings indicated that 29 of the 33 variables had high relative reliability (ICC: 0.508–0.979) and acceptable absolute reliability (CV%: 0.61–14.70), supporting the study hypotheses. The only variables which demonstrated low to moderate relative reliability or unacceptable CV% in the current testing battery were IMTP force at 200 ms, CMJ time to stabilise and landing stiffness, and single leg squat right leg FPPA. Additionally, most metrics were not statistically significantly different between sessions, with trivial to small effect sizes (Table 3, Table 4, Table 5 and Table 6), indicating minimal bias, excluding IMTP force at 150, 200, and 250 ms, CMJ peak landing force, and left 505 completion time, which displayed small, significant improvements in session 2. The favourable results of the current study support the reliability of a testing battery for monitoring and pre-screening female adolescent athletes.

### 5.1. IMTP and CMJ Reliability

The IMTP is considered a more efficient and safer method of assessing strength compared to a one repetition max protocol [35,36]. In a large group setting such as team testing throughout the season, collecting insightful data in a minimal time frame is optimal, hence the IMTP being a preferred method of assessment. When comparing the IMTP peak force results to previous IMTP between-session reliability literature, the ICC and CV% values are similar to those of Moeskops et al.’s [24] post-PHV female gymnast group with almost perfect ICCs and good to excellent CV% values (−0.95; CV%: 5.8–6.7%). Given that Moeskops et al.’s [24] post-PHV group were similar with respect to mean age and maturity offset, the IMTP peak force reliability within post-PHV adolescent females can be considered strong. Furthermore, Thomas et al. [9] also reported similar IMTP peak force between-session reliability measures in adolescent athletes (ICC: 0.95; CV%: 6.11%). It should be noted, however, that Thomas et al.’s [9] population were older, and they pooled their data in a mixed male and female group of adolescent athletes and thus are not fully representative of female adolescent athletes. Corroborating the results of the current study, Thomas et al. [9] also observed non-significant trivial differences in IMTP peak force between sessions. With respect to time-specific force metrics in the present study, the forces at 150, 200, and 250 ms showed statistically significant small increases between sessions in addition to having acceptable CV% values compared to the ‘good’ CV classification given to all the other IMTP measures. These findings from the current study could be due to the participants’ lack of familiarity with the assessment or Olympic weightlifting derivatives. An additional consideration is the sensitivity of time-specific forces (i.e., forces at 50–250 ms) and their ability to be reliably reproduced compared to maximal force. A previous study [55] which observed impulse at the aforementioned time stamps noted a greater variance between sessions (CV%), which is similar to the current findings, with time-specific force values sensitive to initiation detection which can affect the resultant time-specific values [56]. Conducting a third testing session may have resulted in the stabilisation of the time-specific force value; hence, it is a recommended area for further research. Consequently, the IMTP peak force and force at 50 and 100 ms are highly reliable metrics between sessions in female adolescent athletes, which are recommended for longitudinal monitoring of changes in neuromuscular performance in this population. Practitioners should therefore consider changes of 19.7%, 24.4%, and 29.1% in peak force, and force at 50 and 100 ms as meaningful.

The vertical CMJ is an effective method of assessing the slow stretch-shortening cycle neuromuscular function in athletes [39]. Additionally, jump height from this assessment is used as a key performance indicator for sporting and talent recruitment purposes, especially at the university level. Overall, the measures of the current study were found to be reliable with very high to nearly perfect ICCs, trivial to small effect sizes, and no significant differences between sessions except in peak landing force (Table 3). The coefficient of variation was found to be good to excellent in all measures except for time to stabilise and landing stiffness (Table 3). Thomas et al. [9] observed nearly perfect and good to excellent between-session reliability for CMJ RSImod and jump height in male and female adolescent basketballers (ICC: 0.95 and 0.94; CV%: 6.11 and 2.63%). Additionally, Thomas et al. [9] found that the RSImod and jump height were statistically not significant (*p* = 0.132, 0.431) as did the current findings (*p* = 0.027, 0.270). The current study’s ICC and CV% values for the CMJ are also similar to other previous studies [57]; however, it should be noted that the findings of Dugdale et al. [57] involved male athletes ranging from youth to college-aged adults and the athletes from Dugdale and Arthur’s study [57] came from a single sport whereas the current study had athletes from multiple sports. 

Performing vertical jumps on force platforms provides a deeper insight into neuromuscular function using force–time data. Badby et. al [58] observed very high to nearly perfect (ICC: 0.83–0.95) and good to excellent (CV%: 2.90–8.10%) reliability for jump height, RSImod, jump momentum, peak braking force, and time to take off in male youth soccer players. To the authors’ best knowledge, there is no other literature pertaining to the between-session reliability of CMJ time–force metrics in female adolescents, thus inhibiting direct comparisons to previous studies. As such, this study is the first to quantify the between-session reliability of a diverse range of vertical CMJ strategy, outcome, and landing metrics in adolescent females. Landing stiffness and time to stabilise had less favourable reliability outcomes with unacceptable CV% values, which could be attributed to movement variability. It should be noted that although the CV% value for landing stiffness was unacceptable, a very high ICC was reported; in addition, time to stabilise had a moderate ICC, deeming these measures unreliable for the battery. Consequently, for longitudinal monitoring purposes in adolescent female athletes, the changes in jump height (13.4%), RSImod (23.9%), jump momentum (32.1%), peak braking force (22.3%), and time to take off (31.3%) are considered meaningful. 

### 5.2. Hamstring/Knee Flexor Strength and Triple Hop for Distance Reliability

Hamstring strength is a vital aspect of training for females as posterior strength is a key modifiable risk factor for knee injury mitigation [13]. The Nordbord could be a more accessible way to assess hamstring strength compared to the criterion measure of the isokinetic dynamometer [59], yet there is paucity of reliability data for the Nordbord in adolescent females. Previously, Cuthbert et al. [26] and Opar et al. [59] established a very high to nearly perfect between-session reliability for eccentric hamstring strength in female and male adult soccer players, respectively. Specifically, in the present study, good to excellent CV% values and high to nearly perfect ICCs for between-session reliability were observed for eccentric knee flexor strength in female adolescent athletes, with trivial differences observed between sessions. The values of these measures (ICC and CV%) are similar to those observed by Cuthbert et al. [26] in professional adult female soccer players, with an almost perfect ICC (0.901–0.963) for the right and left leg and an excellent CV% (2.89–4.01%). The more favourable reliability score could be due to the sample group being elite athletes with potentially a more extensive training history with Nordic curls. Opar et al. [59] observed the uni- and bilateral average peak forces and reported very high ICCs. Additional literature on eccentric hamstring strength by Bishop et al. [60] and Ferguson et al. [61] also reported high to very high ICCs with a good CV% for uni- and bilateral peak forces during the Nordbord assessment. Although there was a small difference in variability in the current study between left and right peak forces (CV%: 8.28 and 6.30%), this difference could have been due to the participants having a preferred leg for actions in their sport. The high reliability from previous studies [26,59,60,61] and the current study indicates that the Nordbord is a reliable method for assessing lower body posterior strength in adolescent females. Thus, practitioners should consider changes (SDD%) in the left (23.1%), right (17.6%), and total forces (19.2%) during the Nordbord assessment as meaningful. 

In the current study, left and right leg triple hop for distance displayed excellent between-session reliability, with almost perfect ICCs (0.95) and excellent variability (4.08–4.61%). These measures were similar to the reliability measures of previous investigations in adult, recreational, female, and mixed populations [25,62,63,64]. Previous studies [62,63,64] have reported high to near perfect ICC values. Although Kingston et al.’s [64] participants consisted of adult females experiencing patellofemoral pain, their findings still aligned with the current findings. Other studies [62,63] also used an adult population for their participants. Additionally, McCubbine et al. [25] observed very high reliability for left and right triple hop distance (ICC: 0.85 & 0.87) in youth female soccer players. Compared to the current study, McCubbine et al.’s [25] participants consisted of female 9–11 year olds at the elite level at a professional soccer organisation and arguably may have advanced motor control which could explain the very high reliability. As such, given the high reliability of the current study’s findings, this assessment is a suitable indirect measure of horizontal explosive and impulsive capacities of athletes with changes of 11.5% and 11.8% for left and right triple hops for distance, respectively, and can be considered as meaningful for longitudinal monitoring of female adolescent athletes.

### 5.3. 30 m Sprint and 505 COD Speed Reliability

Linear speed and COD are crucial factors for team and individual performance in multiple sports [65,66]. Linear speed assessments are a proxy of fast stretch-shortening cycle function [67], while COD is linked to the directional change requirements associated with numerous sports [65]. The between-session reliability for the 30 m sprint in the current study aligns with that of previous studies [61,68] where there was high to almost perfect ICCs with good to excellent CV% values for split time intervals up to 30 m (Table 5). Although the participants in Ferguson et al.’s [61] and Edward et al.’s [68] research consisted of adolescent males, the present study confirmed that the 30 m sprint is a reliable method of assessing linear sprint speed in adolescent females. As such, changes of 6.2%, 9.3%, and 6.2% should be considered meaningful when longitudinally monitoring changes in 10, 20, and 30 m linear sprint time, respectively, in this population. 

For the 505 COD, the current study reported strong reliability with very high to almost perfect ICCs (0.78–0.95) and excellent CV% values (1.80–2.96%). Comparing Barber et al’s [48] (ICC: 0.965), Dugdale et al’s [57] (ICC: 0.860–0.970), and Taylor et al’s [69] (ICC: 0.260–0.540) findings, the ICC values varied due to their participant demographics as they included youth to adult participants. It should be noted, however, that the 505 time for the left foot was statistically significantly for session 2 with a small improvement. This observation could be due to 90% (n = 28) of the participants preferring their right foot for turning in their sport, and thus there could have been a possible learning effect. Consequently, the present study may have benefitted from a third testing session to establish whether stability in the 505 completion time would be observed. Nonetheless, in context of the present study, the 505 test is a reliable tool for assessing COD speed for both feet in adolescent females, with changes of 6.8% and 7.8% considered to be meaningful for the left and right 505 tests, respectively.

### 5.4. 2D Analysis Reliability

During dynamic sports, poor lower body biomechanics during uni- and bilateral landing and change of directions activities have been identified as primary mechanisms of lower-limb injury, including acute traumatic injuries such as ACL strains or tears or chronic overuse injuries such as patellofemoral pain [70]. For female athletes, the risk of sustaining the aforementioned injuries is higher, due to a multitude of factors such as hormonal, environmental, and anatomical factors [71]; however, biomechanical and neuromuscular control is an important modifiable risk factor which can be addressed through screening and intervention. One such field-based method of lower-limb screening is measuring the FPPA, which can be assessed during a variety of tests such as the single leg squat and tuck jump, to evaluate low- and high-velocity lower-limb control. Previous studies [64,72,73,74] reported very high to almost perfect ICC values (0.700–0.998) compared to the current study, which reported moderate to very high values (ICC: 0.47–0.87) for FPPA in the single leg squat. It should be noted that Simon et al.’s [73] study assessed FPPA using a box step down compared to the other mentioned studies that assessed FPPA using tuck jumps and single leg squats. Based on the current findings, the tuck jump measures were more favourable, with higher ICCs and SEMs (Table 6), and therefore may be considered a better test for assessing lower-limb control in the female adolescent population. Further research in this population for single leg squat FPPA is recommended, but nevertheless, this present study provides SDD% values for monitoring real and meaningful (SDD%) changes for SLSs (right leg: 113.4%; left leg: 115.3%) and tuck jumps (right leg: 88.8%; left leg: 66.6%). 

### 5.5. Limitations

It should be noted that the present study has several limitations. While FPPA has been identified as a surrogate measure for 3D motion knee valgus [44], the 2D video analysis of tasks can be susceptible to parallax errors. To mitigate the potential for parallax to occur within the tuck jump and single leg squat analysis, the cameras were placed as close as reasonably possible to the participants as was performed by Neal and Lack [75], and the athletes were positioned centrally to the camera. Additionally, the study would have also benefitted from a third testing session to establish whether there was stability in some of the measures associated with the tests including the IMTP and 505 left tests, due to the potential learning or familiarisation effects observed within and between sessions [76]. However, the present study provides the day-to-day variability and meaningful changes in an ecologically valid testing battery whilst providing normative data pertaining to female adolescent athletes. Due to legal reasons, the authors were not able to obtain information regarding the menstrual status of the athletes in the current study; however, based on the generally low quality of the evidence, the effect of the menstrual cycle phase and contraception usage has been suggested to have a limited effect on exercise performance and injury risk screening [11,77,78]. The current study only examined sprint and COD times but not instantaneous velocity profiles for sprint and COD; therefore, biomechanical differences could have occurred between sessions. Clarke and Read [76] found that during the different phases of the 505 test (i.e., entry, braking, and exit), variability between each phase during multiple sessions was observed as the athletes found new ways to complete the task. Additionally, the study did not include deceleration measures during the COD trials and did not quantified deceleration ability; thus, this is a recommended area for future research given the importance of deceleration ability in numerous multidirectional sports [79].

## 6. Conclusions

Out of the 33 measures related to athletic performance, neuromuscular function, and injury surrogates, 29 exhibited high to nearly perfect ICC values and 26 were statistically not significantly different between sessions, with acceptable to excellent CV% values and trivial to small effect sizes across all measures. Two measures, landing stiffness and time to stabilise, exhibited unacceptable CV% values. Five measures (left 505 time, CMJ peak landing force, and IMTP force at 150, 200, and 250 ms) were statistically significantly different between sessions. As such, the testing battery can be considered generally highly reliable between sessions for evaluating maximal and rapid lower force production, slow SSC function, horizontal hopping, eccentric knee flexor strength, linear speed, and COD speed, in addition to high-velocity lower-limb control in female adolescent athletes. To the authors’ best knowledge, the present study provides novel reference and normative data for a plethora of tests in adolescent females; thus, practitioners and researchers can use the SDD% values established in the study for longitudinally monitoring changes in performance. 

## Figures and Tables

**Table 1:** Standardised warm-up procedures.

Movement	Sets	Duration/Repetition	Intensity
Hamstring scoops	1	10 ES	
Quad Pulls	1	10 ES	
Hip gates	1	5 ES each direction	
T-lunge	1	5 ES	
Arm circles	1	10 each direction	
Jog	2	15 m	30% (set 1) and 50% (set 2) of maximum perceived effort
Skip	2	15 m	
Side shuffle	2	10 m	
Pogos	1	5 m	

Key: ES = each side; m = metres.

**Table 2:** Force platform testing metrics.

Vertical Countermovement Jump Assessment Measures		
Jump height	Metres (m)	The vertical height achieved by the centre of mass after take off. Calculated from take-off velocity (take-off velocity 2 ÷ 2 g).
Countermovement displacement	Metres (m)	The peak negative vertical displacement of the system centre of mass.
System weight	Newtons (N)	The lowest 1 s average of the vertical ground reaction force applied to the system’s centre of mass during the weighing phase, identified by an optimisation loop.
Jump momentum	Kg × m/s	The vertical momentum of the system centre of mass at the instant of take off.
Peak force	Newtons (N)	Maximum vertical ground reaction force generated during dynamic task from force/time curve.
Time to take off	Seconds (s)	The time it takes for an object or body to leave the ground or surface.
Reactive strength index modified (RSImod)	Arbitrary unit	Performance outcome relative to time in tasks with an identifiable ground contact time. The quotient of dividing jump height by the ground contact time.
Net propulsive Impulse	Newtons per second (Ns)	An amount of force applied for given period of time to cause change in momentum.
Landing stiffness	Newtons (N/m)	The vertical ground reaction force applied to the system’s centre of mass at the instant of peak negative vertical displacement of the system centre of mass divided by the peak negative displacement of the system centre of mass during the landing phase
Time to stabilisation	Milliseconds (ms)	The time taken for the vertical ground reaction forces to the system’s centre of mass to remain within 5% of the weighing system for 1 s.
Average landing force	Newtons (N)	The average vertical ground reaction force applied to the system’s centre of mass during the landing phase
Peak landing force	Newtons (N)	The peak instantaneous vertical ground reaction force applied during the landing phase
Isometric mid-thigh pull assessment measures		
Peak force	Newtons (N)	Maximum vertical ground reaction force generated during isometric task from force/time curve—inclusive of body weight.
Force at specific time (50, 100, 150, 200, and 250 ms)	Newtons (N)	Force at a specific time point from the onset of the contraction (i.e., 0 ms)—inclusive of body weight.

**Table 3:** Between-session reliability for IMTP and CMJ variables.

		Session 1		Session 2		Between-Session Reliability Statistics								
	Metric	Mean	SD	Mean	SD	ICC	CV%	SEM	SDD	SDD%	*p*	ES	Mean Change	SD
IMTP	Peak force (N)	944.37	208.66	970.58	241.20	0.909	7.40	68.03	188.57	19.69	0.270	0.12	26.22	129.96
	Force at 50 (N)	511.47	100.59	525.75	126.91	0.841	9.88	45.66	126.56	24.40	0.356	0.12	14.28	84.89
	Force at 100 (N)	571.21	109.56	608.94	152.64	0.783	10.49	61.89	171.55	29.07	0.064	0.28	37.73	109.15
	Force at 150 (N)	622.04	111.11	687.77	175.16	0.681	12.40	82.84	229.63	35.06	**0.012**	0.45	65.74	137.41
	Force at 200 (N)	688.13	121.23	766.66	203.82	0.546	14.70	112.99	313.19	43.06	**0.023**	0.47	78.52	182.31
	Force at 250 (N)	751.93	137.62	830.27	195.34	0.689	12.25	94.23	261.18	33.02	**0.009**	0.46	78.34	155.77
CMJ	JH (m)	0.24	0.06	0.24	0.06	0.959	4.76	0.01	0.03	13.40	0.419	0.06	0.00	0.02
	Countermovement displacement (m)	−0.23	0.07	−0.22	0.07	0.801	−13.46	0.03	0.09	−39.39	0.148	0.21	0.02	0.06
	System weight (N)	646.47	99.62	640.01	105.01	0.979	1.29	14.83	41.11	6.39	0.230	0.06	−6.46	29.36
	Time to take off (s)	0.73	0.15	0.70	0.14	0.839	7.78	0.06	0.16	22.28	0.120	0.21	−0.03	0.11
	Jump momentum (kg × m/s)	141.91	25.53	145.82	37.54	0.730	4.24	16.68	46.24	32.14	0.471	0.12	3.90	29.74
	Propulsive impulse (Ns)	306.29	73.48	295.19	65.66	0.784	9.95	32.38	89.76	29.85	0.301	0.16	−11.10	58.69
	RSImod	0.34	0.09	0.35	0.09	0.897	7.84	0.03	0.08	23.92	0.373	0.10	0.01	0.06
	Landing stiffness (N)	−8690.86	4668.80	−8827.53	5510.32	0.816	−22.65	2190.63	6072.11	−69.32	0.853	0.03	−136.67	4074.43
	Time to stabilise (ms)	878.28	293.65	993.01	410.42	0.476	21.67	258.31	716.00	76.52	0.135	0.32	114.73	415.77
	Avg landing force (N)	815.24	135.51	805.06	125.23	0.966	3.34	24.06	66.68	8.23	0.239	0.08	−10.17	47.10
	Peak landing force (N)	2396.48	738.94	2203.77	557.04	0.842	10.39	260.10	720.95	31.34	**0.027**	0.29	−192.72	460.72

Key: IMTP = isometric mid-thigh pull; CMJ = countermovement jump; N = Newtons; m = metres; s = seconds; kg × m/s = kilogram × metres per second; Ns = Newton seconds; RSImod = relative strength index modified; ms = milliseconds; SD = standard deviation; ICC = intraclass correlation coefficient; CV% = coefficient of variation; SEM = standard error of measurement; SDD = smallest detectable difference; SDD% = smallest detectable difference as a percentage of the mean; *p* = *p*-value; ES = effect size. Bold denotes significant difference (*p* < 0.05).

**Table 4:** Between-session reliability for hamstring/knee flexor strength and triple hops for distance variables.

		Session 1		Session 2		Between-Session Reliability Statistics								
	Metric	Mean	SD	Mean	SD	ICC	CV%	SEM	SDD	SDD%	*p*	ES	Mean Change	SD
Hamstring/knee flexor strength	Left force (N)	216.36	54.22	224.62	52.44	0.881	8.28	18.40	51.00	23.13	0.192	0.15	8.25	34.44
	Right force (N)	214.27	52.95	218.05	48.63	0.927	6.30	13.74	38.08	17.61	0.437	0.07	3.78	26.72
	Total force (N)	430.63	105.40	442.66	99.32	0.913	6.91	30.21	83.73	19.18	0.254	0.12	12.04	57.64
Triple hops for distance	Right distance (m)	3.74	0.75	3.84	0.71	0.954	4.08	0.16	0.44	11.50	0.070	0.14	0.10	0.30
	Left distance (m)	3.77	0.72	3.89	0.67	0.945	4.61	0.16	0.45	11.83	0.303	0.18	0.12	0.30

Key: N = Newtons; m = metres; SD = standard deviation; ICC = intraclass correlation coefficient; CV% = coefficient of variation as a percentage; SEM = standard error of measurement; SDD = smallest detectable difference; SDD% = smallest detectable difference as a percentage of the mean; *p*= *p*-value; ES = effect size.

**Table 5:** Between-session reliability for 30 m sprint and 505 change of direction speed variables.

		Session 1		Session 2		Between-Session Reliability Statistics								
	Metric	Mean	SD	Mean	SD	ICC	CV%	SEM	SDD	SDD%	*p*	ES	Mean Change	SD
30 m sprint	Time at 10 m (s)	2.16	0.14	2.16	0.14	0.887	2.14	0.05	0.13	6.16	0.852	0.02	0.00	0.09
	Time at 20 m (s)	3.78	0.30	3.76	0.27	0.809	2.55	0.13	0.35	9.32	0.696	0.06	−0.02	0.23
	Time at 30 m (s)	5.31	0.42	5.36	0.45	0.926	2.07	0.12	0.33	6.15	0.223	0.12	0.05	0.23
Left foot 505	10 m approach (s)	2.21	0.15	2.20	0.16	0.946	1.80	0.04	0.10	4.51	0.738	0.03	0.00	0.07
	505 time (s)	2.80	0.17	2.75	0.18	0.852	2.37	0.07	0.19	6.83	**0.023**	0.29	−0.05	0.12
Right foot 505	10 m approach (s)	2.21	0.17	2.22	0.17	0.911	2.23	0.05	0.14	6.24	0.941	0.01	0.00	0.10
	505 time (s)	2.79	0.17	2.74	0.17	0.782	2.96	0.08	0.22	7.79	0.077	0.27	−0.05	0.14

Key: s = seconds; 505 time = (10 m approach − completion time); SD = standard deviation; ICC = intraclass correlation coefficient as a percentage; CV% = coefficient of variation; SEM = standard error of measurement; SDD = smallest detectable difference; SDD% = smallest detectable difference as a percentage of the mean *p* = *p*-value; ES= effect size. Bold denotes significant difference (*p* < 0.05).

**Table 6:** Between-session reliability for tuck jump and single leg squat FPPA measures.

		Session 1		Session 2		Between-Session Reliability Statistics							
	Metric	Mean	SD	Mean	SD	ICC	SEM	SDD	SDD%	*p*	ES	Mean Change	SD
Tuck Jump FPPA (deg.)	R. leg FPPA	−10.39	7.62	−12.06	9.35	0.822	3.60	9.97	−88.84	0.165	0.20	−1.67	6.55
	L. leg FPPA	−16.83	10.90	−16.10	11.38	0.874	3.96	10.97	−66.62	0.596	0.07	0.72	7.53
Single Leg Squat FPPA (deg.)	R. leg FPPA	−17.71	10.19	−17.43	9.45	0.465	7.19	19.92	−113.40	0.895	0.03	0.28	11.66
	L. leg FPPA	−17.39	10.68	−17.40	9.94	0.508	7.24	20.06	−115.33	0.996	0.00	−0.01	11.92

Key: R = right; L = left; FPPA = (knee angle – 180); SD = standard deviation; ICC = intraclass correlation coefficient; CV% = coefficient of variation as a percentage; SEM = standard error of measurement; SDD = smallest detectable difference; SDD% = smallest detectable difference as a percentage of the mean; *p* = *p*-value; ES = effect size.

## Data Availability

The raw data supporting the conclusions of this article will be made available by the authors on request.

## Supplementary Materials

The following supporting information can be downloaded at: https://www.mdpi.com/article/10.3390/life14070892/s1, Table S1: Confidence intervals of the ICC and CV% values for the IMTP and CMJ; Table S2: Confidence intervals of the ICC and CV% values for hamstring/knee flexor strength and triple hops for distance; Table S3: Confidence intervals of the ICC and CV% values for the 30 m sprint and 505 change of direction; Table S4: Confidence intervals of tuck jump and single leg squat FPPAs; Table S5: Within-session reliability for IMTP and CMJ variables; Table S6: Within-session reliability of hamstring/knee flexor strength variables; Table S7: Within-session reliability of 30 m sprint and 505 change of direction variables; Table S8: Within-session reliability of tuck jump and single leg squat FPPA measures.
