# Supplementary material for: Between-Session Reliability of Athletic Performance and Injury Mitigation Measures in Female Adolescent Athletes in the United States
Source: Life (Basel). 2024 Jul 18;14(7):892. doi: 10.3390/life14070892 (PMC11278427; doi:10.3390/life14070892)
Supplement: Supplementary file 1 [file life-14-00892-s001.zip › life-3093410-supplementary.pdf]

Supplementary Materials

Table S1. Confidence Intervals of the ICC and CV% values of the IMTP and CMJ.

	**Metric**	**ICC**	**LB**	**UB**	**CV%**	**LB**	**UB**
**IMTP**	Peak force (N)	0.909	0.812	0.956	7.40	4.98	9.83
	Force at 50 (N)	0.841	0.673	0.923	9.88	6.74	13.02
	Force at 100 (N)	0.783	0.552	0.895	10.49	7.20	13.77
	Force at 150 (N)	0.681	0.329	0.848	12.40	8.91	15.90
	Force at 200 (N)	0.546	0.098	0.776	14.70	10.39	19.00
	Force at 250 (N)	0.689	0.335	0.835	12.32	9.21	15.43
**CMJ**	JH (m)	0.957	0.838	0.912	4.76	2.99	4.76
	Countermovement displacement (m)	0.801	0.591	0.903	-13.46	-18.29	-13.46
	System weight (N)	0.979	0.956	0.990	0.61	0.45	0.61
	Time to take off (s)	0.839	0.668	0.922	7.78	5.13	7.78
	Jump momentum (kg*m/s)	0.730	0.440	0.870	2.55	1.61	2.55
	Propulsive impulse (Ns)	0.784	0.556	0.895	9.95	5.84	9.95
	RSImod	0.897	0.788	0.950	7.84	5.46	7.84
	Landing stiffness (N)	0.816	0.615	0.911	-22.65	-27.96	-22.65
	Time to stabilize (ms)	0.476	-0.054	0.743	21.67	12.52	21.67
	Avg landing force (N)	0.966	0.930	0.983	3.34	2.37	3.34
	Peak landing force (N)	0.842	0.656	0.925	10.39	7.19	10.39

Key: IMTP= isometric mid-thigh pull; CMJ= countermovement jump; N = Newtons; m = metres; s = seconds; kg*m/s= kilogram * metres per second; Ns= Newton seconds; RSImod = relative strength index modified; ms = milliseconds; SD= standard deviation; ICC= intraclass correlation coefficient; CV%= coefficient of variation; LB= lower bound; UB= upper bound

Table S2. Confidence Intervals of the ICC and CV% Values Hamstring/Knee Flexor Strength and Triple Hops for Distance.

	Metric	ICC	LB	UB	CV%	LB	UB
**Hamstring/knee flexor strength**	Left force (N)	0.881	0.756	0.942	8.28	5.22	8.28
	Right force (N)	0.927	0.849	0.965	6.30	4.09	6.30
	Total force (N)	0.913	0.821	0.958	6.91	4.46	6.91
**Triple hops for distance**	Right distance (m)	0.954	0.903	0.978	0.95	2.62	4.08
	Left distance (m)	0.945	0.876	0.974	0.95	2.99	4.61

Key: N= Newtons; m = metres; ICC= intraclass correlation coefficient; CV%= coefficient of variation as a percentage; LB= lower bound; UB= upper bound

Table S3. Confidence Intervals of the ICC and CV% Values of the 30m sprint and 505 Change of Direction.

	**Metric**	**ICC**	**LB**	**UB**	**CV%**	**LB**	**UB**
**30 m Sprint**	Time at 10 m (s)	0.887	0.765	0.946	2.14	1.40	2.14
	Time at 20 m (s)	0.809	0.603	0.908	2.55	1.39	2.55
	Time at 30 m (s)	0.926	0.848	0.964	2.07	1.29	2.07
**Left foot 505**	10 m approach (s)	0.946	0.887	0.974	1.80	1.33	1.80
	505 time (s)	0.852	0.674	0.930	2.37	1.54	2.37
**Right foot 505**	10 m approach (s)	0.911	0.816	0.957	2.23	1.56	2.23
	505 time (s)	0.782	0.552	0.895	2.96	2.27	2.96

Key: s = seconds; 505 time = (10m approach – completion time); ICC= intraclass correlation coefficient as a percentage; CV%= coefficient of variation; LB= lower bound; UB= upper bound

Table S4. Confidence Intervals of Tuck Jumps and Single Leg Squat FPPA.

	Metric	ICC	LB	UB
**Tuck jump FPPA (deg.)**	R. leg FPPA	0.822	0.636	0.914
	L. leg FPPA	0.874	0.738	0.939
**Single leg squat FPPA (deg.)**	R. leg FPPA	0.465	-0.131	0.744
	L. leg FPPA	0.508	0.738	0.939
	Key: R = right; L = Left; FPPA = (Knee angle – 180); ICC= intraclass correlation coefficient; LB= lower bound; UB= upper bound			

Table S5. Within Session Reliability for IMTP and CMJ Variables.

		Session 1					Session 2				
	Metric	Mean	SD	ICC	CV%	SEM	Mean	SD	ICC	CV%	SEM
**IMTP**	Peak force (N)	944.37	208.66	0.890	4.76	67.97	970.58	241.20	0.976	4.88	38.75
	Force at 50 (N)	511.47	100.59	0.789	5.03	48.00	525.75	126.91	0.961	6.57	25.96
	Force at 100 (N)	571.21	109.56	0.711	8.22	64.67	608.94	152.64	0.951	7.49	36.09
	Force at 150 (N)	622.04	111.11	0.715	9.04	67.86	687.77	175.16	0.958	7.28	37.83
	Force at 200 (N)	688.13	121.23	0.711	8.91	73.53	766.66	203.82	0.949	8.96	45.14
	Force at 250 (N)	757.40	121.23	0.744	7.80	77.93	830.27	203.82	0.958	6.38	42.24
		Session 1					Session 2				
**CMJ**	JH (m)	0.24	0.06	0.979	4.95	0.01	0.24	0.06	0.977	4.67	0.01
	Countermovement displacement (m)	-0.23	0.07	0.965	10.01	0.01	-0.22	0.07	0.953	11.99	0.02
	System weight (N)	646.47	99.62	1.000	0.05	0.00	645.05	98.73	1.000	0.09	0.00
	Time to take off (s)	0.73	0.15	0.949	7.07	0.03	0.70	0.14	0.879	10.02	0.05
	Jump momentum (kg*m/s)	141.91	25.53	0.993	2.28	2.18	140.65	25.59	0.991	2.37	2.46
	Propulsive impulse (Ns)	306.29	73.48	0.954	6.92	15.59	295.19	65.66	0.987	3.75	7.63
	RSImod	0.34	73.48	0.944	7.56	0.02	0.35	65.66	0.905	11.50	0.03
	Landing stiffness (N)	-8690.86	4668.80	0.834	28.53	2082.76	-8827.53	5510.32	0.830	24.20	2652.35
	Landing stiffness (N)	-8690.86	4668.80	0.834	28.53	2082.76	-8827.53	5510.32	0.830	24.20	2652.35
	Time to stabilise (ms)	885.81	285.63	0.667	22.29	221.72	993.01	410.42	0.618	20.56	274.76
	Average landing force (N)	815.24	135.51	0.969	4.16	24.34	805.06	125.23	0.950	4.29	38.03
	Peak landing force (N)	2396.48	738.94	0.873	15.17	288.99	2203.77	557.04	0.876	12.83	262.26
	Key: IMTP= isometric mid-thigh pull; CMJ= countermovement jump; N = Newtons; m = metres; s = seconds; kg*m/s= kilogram * metres per second; Ns= Newton seconds; RSImod = relative strength index modified; ms = milliseconds; SD= standard deviation; ICC= intraclass correlation coefficient; CV%= coefficient of variation; SEM= standard error of measurement										

Table S6. Within Session Reliability of Hamstring/Knee Flexor Strength Variables.

		Session 1					Session 2				
	Metric	Mean	SD	ICC	CV%	SEM	Mean	SD	ICC	CV%	SEM
**Hamstring/knee flexor strength**	Left peak force (N)	216.36	54.22	0.967	7.21	10.63	224.62	52.44	0.936	9.02	13.78
	Right peak force (N)	214.27	52.95	0.963	7.13	11.13	218.05	48.63	0.958	6.38	10.54
	Total force (N)	430.63	105.40	0.970	6.51	19.70	442.66	99.32	0.958	6.77	21.03
		Session 1					Session 2				
**Triple hops for distance**	Left distance (m)	3.74	0.75	0.948	6.36	0.17	3.84	0.71	0.971	5.01	0.12
	Right distance (m)	3.77	0.72	0.959	5.48	0.15	3.89	0.67	0.98	5.43	0.10
	Key: N= Newtons; m = metres; SD= standard deviation; ICC= intraclass correlation coefficient; CV%= coefficient of variation as a percentage; SEM= standard error of measurement										

Table S7. Within Session Reliability of 30 m Sprint and 505 Change of Direction Variables.

		Session 1					Session 2				
	Metric	Mean	SD	ICC	CV%	SEM	Mean	SD	ICC	CV%	SEM
**30m Sprint**	10m time (s)	2.16	0.14	0.852	2.37	0.06	2.16	0.14	0.947	2.16	0.03
	20m time (s)	3.74	0.26	0.899	1.87	0.09	3.76	0.27	0.978	1.56	0.04
	30m time (s)	5.31	0.42	0.953	1.74	0.09	5.36	0.45	0.978	1.70	0.07
		Session 1					Session 2				
**Left foot 505**	10 m approach (s)	2.21	0.15	0.957	1.97	0.03	2.20	0.16	0.963	2.01	0.03
	505 time (s)	2.80	0.17	0.698	3.36	0.13	2.75	0.18	0.95	2.14	0.04
		Session 1					Session 2				
**Right foot 505**	10 m approach (s)	2.21	0.17	0.950	2.35	0.04	2.22	0.17	0.972	1.82	0.03
	505 time (s)	2.79	0.17	0.921	2.58	0.05	2.74	0.17	0.929	2.33	0.05

Key: s = seconds; 505 time = (10m approach – completion time); SD= standard deviation; ICC= intraclass correlation coefficient as a percentage; CV%= coefficient of variation; SEM= standard error of measurement

Table S8. Within Session Reliability of Tuck Jump and Single Leg Squat FPPA Measures.

		Session 1				Session 2			
**Tuck jumps FPPA (deg.)**	Metric	Mean	SD	ICC	SEM	Mean	SD	ICC	SEM
	R. leg FPPA	-10.39	7.62	0.769	4.23	-12.06	9.35	0.851	4.13
	L. leg FPPA	-16.72	11.04	0.902	3.76	-16.10	11.38	0.874	4.90
**Single leg squat FPPA (deg.)**	R. leg FPPA	-17.96	10.08	0.901	3.28	-17.22	9.79	0.86	4.03
	L. leg FPPA	-17.13	10.77	0.904	3.78	-17.40	9.94	0.821	5.04

Key: R = right; L = Left; FPPA = (Knee angle – 180); SD= standard deviation; ICC= intraclass correlation coefficient; CV%= coefficient of variation as a percentage; SEM= standard error of measurement
